# Cytoskeleton proteins previously considered exclusive to Ganglion Cells are transiently expressed by all retinal neuronal precursors

**DOI:** 10.1186/1471-213X-11-46

**Published:** 2011-07-22

**Authors:** Christian Gutierrez, Minda McNally, M Valeria Canto-Soler

**Affiliations:** 1 Wilmer Eye Institute, Department of Ophthalmology, Johns Hopkins University School of Medicine, 400 N Broadway, Baltimore, MD USA

## Abstract

**Background:**

Understanding the mechanisms governing cell fate specification remains one of the main challenges in the study of retinal development. In this context, molecular markers that identify specific cell types become crucial tools for the analysis and interpretation of these phenomena. In studies using the developing chick retina, expression of the mid-size neurofilament (NF-M) and a chick-specific microtubule associated protein recognized by the RA4 antibody (MAP(RA4)), have been broadly used to selectively identify ganglion cells and their committed precursors. However, observations in our laboratory suggested that the expression of these proteins may not be restricted to cells of the ganglion cell lineage. Because of its potential significance in the field, we pursued a detailed analysis of the expression of these two molecules in combination with an array of proteins that allowed precise identification of all retinal cell-type precursors throughout the development of the chick retina.

**Results:**

Both, NF-M and MAP(RA4) proteins, showed a dynamic pattern of expression coincident with the progression of retinal cell differentiation. Both proteins were coexpressed spatially and temporally in postmitotic neuronal precursors throughout development. Expression of both proteins was seen in ganglion cell precursors and adult differentiated ganglion cells, but they were also transiently expressed by precursors of the photoreceptor, horizontal, bipolar and amacrine cell lineages.

**Conclusions:**

We have clearly demonstrated that, contrary to the generally accepted paradigm, expression of NF-M and MAP(RA4) proteins is not exclusive to ganglion cells. Rather, both proteins are transiently expressed by all neuronal retinal progenitors in a developmentally-regulated manner. In addition, MAP(RA4) and NF-M are the first molecules so far characterized that may allow unambiguous identification of postmitotic precursors from the pool of mitotically active progenitors and/or the differentiated cell population during retinogenesis. These results are of significant impact for the field of developmental biology of the retina, since they provide novel and important information for the appropriate design and interpretation of studies on retinal cell differentiation, as well as for the reinterpretation of previously published studies.

## Background

The mature retina consists of five major neuronal cell types including photoreceptors, horizontal, bipolar, amacrine and ganglion cells, and the glial cells of Müeller. These cells are mitotically quiescent (i.e., "postmitotic"), and can be distinguished from each other by their shape, molecular composition, function, and location in the characteristic layers of the retina. These highly diverse cell types originate during normal development from a morphologically homogeneous, mitotically active population of retinal progenitor cells [1]. The elucidation of the mechanisms controlling this complex process of cell differentiation has for decades attracted the interest of developmental neurobiologists, but despite this effort, they still remain mostly unknown.

In the chick, the neuronal elements of the retina are born between embryonic day (ED) 3 and ED8 in the central region of the embryonic eye ([1] and references therein). During this period, cell proliferation, cell birth (i.e., the permanent withdrawal of individual cells from the mitotic cycle), cell migration and cell differentiation occur mostly in an overlapping manner ([1] and references therein). A consequence of this chronology is that, at the stages frequently used in experiments aimed at analyzing mechanisms of cell differentiation (e.g., ED 6-8), the retina contains a mixture of proliferating cells that are at different phases of the cell cycle, and postmitotic cells that are at different stages of migration to their definitive laminar positions and/or have reached various degrees of differentiation [1]. Therefore, not only the identification of genes that are expressed in a cell-type specific manner, but also the identification of genes that may allow differentiating between the pool of postmitotic neuronal precursors and the proliferating cell population at these developmental stages is of significant importance.

The cytoskeleton plays an essential role in processes directly associated with cell differentiation, such as regulation of cell cycle, cell morphology and migration. Initiation of synthesis of the cell-type-specific intermediate filament proteins often accompanies the emergence of definitive cell types during embryonic development. In the specific case of neuronal cells, one of the earliest recognizable events in the differentiation of postmitotic neuroblasts is the appearance of neurofilament proteins [2-4]. In birds and mammals neurofilaments are composed of three individual proteins of different molecular weight: neurofilament-low (NF-L; 68-70 kDa), neurofilament-medium (NF-M; 145-160 kDa), and neurofilament-high (NF-H; 180-210 kDa) [4-6]. Another group of important elements of the cytoskeleton are microtubule-associated proteins (MAPs), whose main function is to regulate microtubule polymerization and stabilization [7], but are also known to interact with neurofilaments in the regulation of mechanisms of cell differentiation [4,8]. The expression of the different neurofilaments and MAPs in different cell types (i.e. neurons vs. non-neurons), their specific subcellular localization, and their developmentally regulated expression reflect their important role during cell differentiation in general and neuronal differentiation in particular [9,10]. Furthermore, their characteristic pattern of expression suggests that they could be useful in the identification of different cell types, particularly at early stages of development when markers of this kind are very limited. In the chick retina, several previous publications have reported expression of the NF-M protein and a specific MAP in a subpopulation of retinal cells. Initial characterization of the monoclonal antibody RA4, which binds to a chick-specific MAP, and studies done at similar stages of development with an antibody that recognizes the phosphorylated form of the chick NF-M, suggested that both proteins were exclusively expressed in committed ganglion cell precursors and adult differentiated ganglion cells [2,11-15].

In this study however, we demonstrate that both, NF-M and the MAP protein recognized by the RMO270 and RA4 antibodies respectively, are not exclusive to cells belonging to the ganglion cell lineage. Rather, both proteins are transiently expressed by all neuronal retinal progenitors in a developmentally-regulated manner. Our observations suggest that initial expression of these molecules may be important in all retinal neuronal cell types for mechanisms regulating proper cell differentiation such as migration, cell morphology and axonal growth, while high sustained levels of expression may be necessary for overt differentiation of long-projecting axon cell types including ganglion cells (GC) and bullwhip cells, and for maintenance of the axonal cytoskeleton network in their adult state. Our findings also demonstrate that MAP(RA4) and NF-M may be useful for unambiguous identification of postmitotic precursors from the pool of mitotically active progenitors and/or the differentiated cell population during retinogenesis. The implications of these results in previously published studies are also discussed.

## Methods

### Animals

All procedures were performed in accordance with the animal protocols approved by the Animal Care and Use Committee at the Johns Hopkins University. Fertilized White Leghorn chicken eggs were obtained from B&E Eggs (York Springs, Pennsylvania). Eggs were incubated at 37.5°C and 60% relative humidity and staged as in [16].

### Immunohistochemistry

Chick embryos were euthanized by decapitation before ED10, and by halothane overdose thereafter. Whole heads were fixed at ED3-8, and enucleated eyes (devoid of cornea and lens) were fixed for later stages. Samples were fixed in 4% paraformaldehyde for 1 hour, washed in PBS (2 × 5 min), and cryoprotected with a sucrose gradient (6.75%, 12.5%, and 25%, overnight at 4°C each) and a final incubation in 25% sucrose/OCT (2:1 ratio respectively) for 1 hour at RT. Samples were embedded in 25% sucrose/OCT Tissue-Tek (Sakura), frozen and stored at -80°C until used. Samples were sectioned from ventral to dorsal and sections (10 μm unless otherwise stated) corresponding to the central region of the eye (50-600 μm from the optic nerve head for ED6) were collected on Superfrost Plus slides. Sections were air dried for 1 hour, washed in PBS (3 × 5 min), blocked in 10% goat serum in PBS with 0.25% TritonX for 1 hour at RT, and incubated overnight with a primary antibody in 2% goat serum in PBS with 0.05% TritonX at 4°C. The next day, the slides were washed in PBS (3 × 5 min) and incubated with an Alexa Fluor-conjugated secondary antibody (1:1000; Molecular Probes) in PBS for 1 hour in the dark at RT. The slides were then washed in PBS (3 × 5 min), incubated in DAPI (1:1000 in PBS) for 10 min, and cover-slipped using DAKO fluorescent mounting medium. Double immunostaining involving RA4 or RMO270 and a second monoclonal primary antibody (Lim3, Lim1/2, Visinin, Vimentin, AP2α, Hu C/D, or Islet1; all from NICHD-funded Developmental Studies Hybridoma Bank maintained by The University of Iowa, Iowa City, IA; see Table 1 for complete antibody information) was performed using tyramide amplification (TSA-PLUS Fluorescein System; Perkin Elmer, Boston, MA Perkin Elmer) as described in [17]. Briefly, sections were blocked and permeabilized in 10% goat serum/0.25% Triton X-100 in PBS for 1 hour at RT, and incubated overnight at 4°C in primary antibody (RMO270, RA4, or Islet1; Table 1) diluted in 2% goat serum/0.05% Triton X-100 in PBS. Antibody binding was detected with sequential incubations with biotinylated goat anti-mouse (1:1000), horseradish peroxidase-streptavidin (1:1000) and fluorescein-tyramide (1:100). Control reactions showed that, at the concentrations indicated in Table 1, these antibodies were detectable after tyramide amplification but undetectable with fluorescent secondary antibodies. Sections were then profusely washed in PBS, followed by overnight incubation with antibodies diluted in PBS (Lim1 and Lim3) or diluted in 2% goat serum/0.05% Triton X-100 in PBS (all other antibodies found in Table 1) and visualized with Alexa Fluor 647 goat anti-mouse secondary antibody. For colocalization studies of the RA4 and RMO270 antibodies, the same approach described above was followed, but RA4 was routinely applied first and developed by the tyramide reaction, followed by RMO270 and Alexa Fluor 647 incubation. Triple immunostaining involving RMO270 and primary antibodies Prox1 and AP2α was performed similarly as double immunostaining for RMO270 and Ap2α, followed by incubation and regular fluorescence detection for Prox1. Images were taken using a Zeiss LSM 510 confocal microscope.

**Table 1:** List of antibodies used in this study

Antibody	Antigen	Cell Type	Species	Indirect Fluorescent	Tyramide Amplification	Source
**RA4**	Microtubule Associated Protein (MAP)	Postmitotic Neuronal Precursors Ganglion Cell axons	Mouse	ED3 1:500	ED3, ED6, ED7 1:10,000 ED8-ED18 1:2000	Kind gift from Dr. Steven McLoon
**RMO270**	Mid-sized Neurofilament (NF-M)	Postmitotic Neuronal Precursors Adult Ganglion and Bullwhip Cells	Mouse	ED3-ED6 1:10,000 ED7-ED18 1:5,000	ED6 1:20,000 ED7-ED18 1:10,000	Kind gift from Dr. Virgnia Lee
**3B5**	AP2α	Amacrine and Horizontal Cells	Mouse	1:35	N/A	Developmental Studies Hybridoma Bank
**Prox1**	Prox1	Horizontal and Bipolar Cells	Rabbit	1:5,000	1:5,000	Millipore
**4F2**	Lim1/2	Horizontal Cells	Mouse	1:5	N/A	Developmental Studies Hybridoma Bank
**67.4E12**	Lim3	Bipolar Cells	Mouse	1:5	N/A	Developmental Studies Hybridoma Bank
**Visinin**	Visinin	Photoreceptor Cells	Rabbit	1:10,000	N/A	Kind gift from Dr. Leveillard
**H5**	Vimentin	Müller Glial Cells	Mouse	1:100	N/A	Developmental Studies Hybridoma Bank
**39.3F7**	Islet1	ED3-ED6: Ganglion Cells ED7 and after: All Retinal Neuronal Cells	Mouse	1:50	1:5,000	Developmental Studies Hybridoma Bank
**Hu C/D**	Hu C/D	ED3-ED6: Ganglion Cells ED7 and after: Ganglion and Amacrine Cells	Mouse	1:100	N/A	Invitrogen

### EdU Incorporation

Click-iT EdU imaging kit was used to visualize cells that have gone through S-phase during the time-window under study [18]. A small window was made on the eggshell of ED3 chick embryos and the chorion and amnion were cut to expose the head of the embryo (stage 21). Fifty micrograms of EdU diluted in PBS were delivered over the embryo's head. The small window on the shell was covered with a strip of tape (3 M Transpore Surgical Tape) and eggs were placed back into the incubator. After five hours the heads were collected and processed for immunohistochemistry as described above. After immunohistochemical detection with RA4 or RMO270 antibodies, the Click-iT EdU imaging kit was used according to the manufacturer's protocol. One micrometer optical thickness images were taken using a Zeiss LSM 510 confocal microscope (n = 3 embryos; 6 sections/embryo). A single image of the complete central portion of the retina containing RA4 or RMO270 positive cells was taken for every section. RA4 and RMO270 positive cells were manually counted to determine the location and percentage of cells positive for these markers and that were also positive for EdU.

## Results

### MAP(RA4) expression pattern in the chick neural retina throughout development

**Figure 1:**
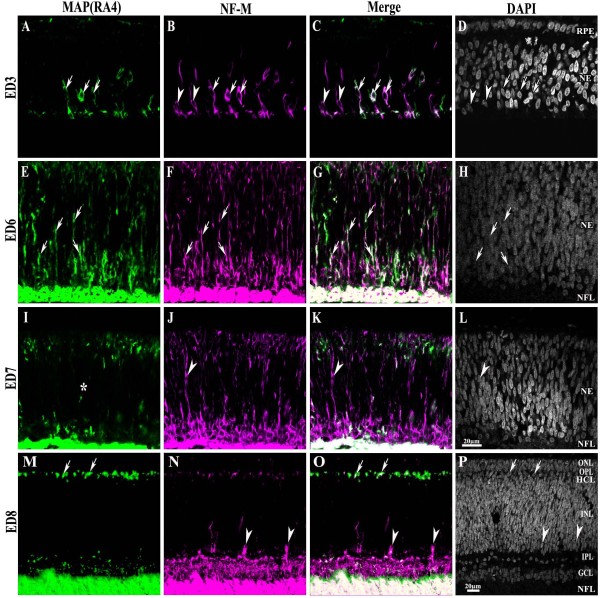
**MAP(RA4) and NF-M expression in the central retina throughout development**. Transverse sections of central chick retina at different stages of development processed for double-label immunohistochemistry for MAP(RA4) (green) and NF-M (magenta). Retinal sections are shown with the pigment epithelium at the top and the vitreal surface of the retina at the bottom. (A-D) At ED3 both MAP(RA4) and NF-M are coexpressed in cells located in the most central region of the retina (arrows); a few cells expressing only NF-M were seen at the leading edge of the neurogenic front (arrowheads). (E-H) At ED6 most if not all MAP(RA4) cells are also positive for NF-M. (I-L) By ED7 MAP(RA4) expression is mostly absent from cells in the middle portion of the retina (I; asterisk) while NF-M remains present (J). (M-P) At ED8 expression of both becomes restricted to the nerve fiber layer, GCL, IPL and HCL, with NF-M being also expressed in the bullwhip cells (arrowheads). Arrows indicate cells that are positive for both markers and arrowheads represent cells that are NF-M positive only. Scale bar in L applies to A-L and scale bar in P to M-P. ED: embryonic day; GCL: ganglion cell layer; HCL: horizontal cell layer; INL: inner nuclear layer; IPL: inner plexiform layer; NE: neuroepithelium; NFL: nerve fiber layer; ONL: outer nuclear layer; OPL: outer plexiform layer; RPE: retina pigmented epithelium.

The RA4 antibody labels a 140 kDa microtubule associated protein (MAP) expressed in long axon projecting neurons in the adult chick [11,19]. Therefore, when describing the expression pattern of this protein we will refer to it as MAP(RA4). MAP(RA4) labeling was seen at all developmental stages examined, and showed a dynamic pattern coincident with the progression of retinal development. In agreement with previous reports [19], MAP(RA4) showed its characteristic "radial" pattern of expression that demarcates the progressive advance of the "neurogenic front" (region containing differentiating neuronal precursors) from central to more peripheral regions. At ED3, MAP(RA4)-radially oriented cells spanning the thickness of the retina were confined to a discrete region of the central retina and their nuclei were found variably positioned throughout the width of the retinal neuroepithelium (Figure 1, A and Additional File 1 Fig S1, A). A similar expression pattern, although with increasing number of positive cells and expanding towards the periphery as development progressed, was observed at ED4-ED5 (not shown) and ED6 (Figure 1, E and Additional File 1, Fig S1, B). After ED6, MAP(RA4) expression pattern changed substantially. As development progressed, and coincident with the advance of the neurogenic front towards more peripheral regions, there was a significant decrease in MAP(RA4)-radially oriented cells in the central retina while still present in more peripheral regions. At ED7, only a few MAP(RA4)-radially oriented cells were observed in the central portion of the retina (Figure 1, I) and by ED8, the central retina was completely devoid of "radial" MAP(RA4) cells; instead, MAP(RA4) labeling was restricted to cells located in the horizontal cell layer (HCL), and neuronal projections in the inner plexiform layer (IPL), the ganglion cell layer (GCL), and the nerve fiber layer (NFL) (Figure 1, M and Additional File 1, Fig S1, c2). As development progressed, MAP(RA4) signal became even more restricted, being present only in cell processes in the IPL and the axons of the GCL, a pattern that was maintained in the adult retina (not shown).

### MAP(RA4) cells expressed the 160 kDa neurofilament protein

**Figure 2:**
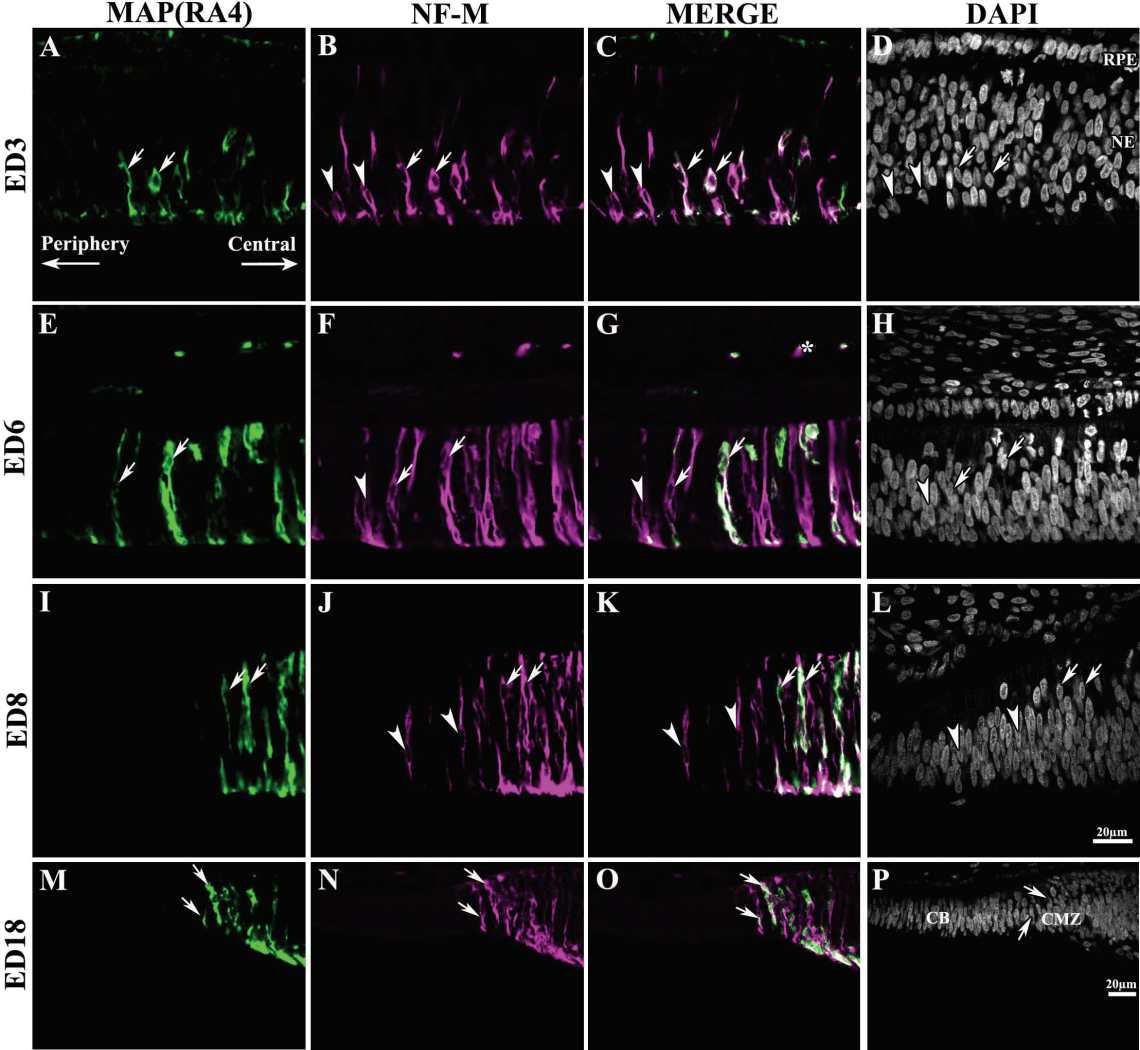
**Progression of MAP(RA4) and NF-M expression towards the periphery during retinal development**. Transverse sections of peripheral regions of chick retina at different stages of development processed for double-label immunohistochemistry for MAP(RA4) (green) and NF-M (magenta). Retinal sections are shown with the pigment epithelium at the top and the vitreal surface of the retina at the bottom. As development progresses MAP(RA4) and NF-M expression expands towards more peripheral regions of the retina until they reach the CMZ. At developmental stages between ED3 and ED8 (A-L) NF-M expression was always seen ahead of that of MAP(RA4). By ED18 (M-P) MAP(RA4) is expressed in most if not all cells expressing NF-M in the most peripheral retina. Arrows indicate cells colabeled with MAP(RA4) and NF-M, and arrowheads point at cells that are only positive for NF-M. Asterisks in E-G indicate transverse-sectioned nerve fibers labeled with MAP(RA4) and NF-M. Scale bar in L applies to A-L and scale bar in P applies to M-P. CB: ciliary body; CMZ: ciliary marginal zone; ED: embryonic day; NE: neuroepithelium; RPE: retina pigmented epithelium.

At all the stages analyzed, a similar expression pattern to that of MAP(RA4) was observed with an antibody against the 160 kDa neurofilament-medium (NF-M) protein (Additional File 1, Fig S1). Furthermore, at all stages, all "radial" MAP(RA4) cells were also positive for NF-M (Figure 1, A-L). However, some differences were observed between the expression pattern of NF-M and that of MAP(RA4). Interestingly, we observed a subpopulation of cells that expressed NF-M but that were negative for MAP(RA4). Most noticeably, NF-M(+)/MAP(RA4)(-) cells were mainly observed at the leading edge of the neurogenic front (Figure 1, A-D and Figure 2; arrowheads). NF-M expression was consistently ahead of MAP(RA4) until ED18, when positive labeling for both reached the ciliary marginal zone (CMZ; Figure 2, M-P). In addition, we also observed that at ED7, when MAP(RA4) radial cells start to disappear from the central retina, a significant number of radially oriented NF-M(+)/MAP(RA4)(-) cells are still distinguishable (Figure 1, I-L); these cells are no longer observed in the central retina at ED8 or thereafter.

As in the case of MAP(RA4), as development progressed expression of the NF-M protein became restricted to the HCL, IPL, GCL and the NFL (ED8; Figure 1, M-P) and disappeared from the HCL at later stages (not shown). However, unlike MAP(RA4), at these stages NF-M expression was also detected in the cell bodies of ganglion cells and in cells located at the inner edge of the amacrine cell layer, the bullwhip cells [20,21] (Figure 1, M-P; arrowheads). This pattern of expression was maintained in the adult retina (not shown).

### MAP(RA4) and NF-M are expressed in postmitotic precursors of all neuronal lineages in the retina

Our observations did not appear consistent with previous reports stating that both, NF-M and MAP(RA4) proteins are exclusively expressed in differentiating and mature ganglion cells [2,11-15,22-24]. In contrast, the patterns of expression observed for MAP(RA4) and NF-M are compatible with two other possible scenarios: i) the NF-M protein is expressed in all retinal neuronal precursors as they begin to differentiate, while MAP(RA4) is expressed only in a subpopulation of these precursors corresponding to the ganglion cell lineage; ii) NF-M and MAP(RA4) are expressed in all retinal neuronal precursor cell types, with NF-M being expressed first and soon after followed by MAP(RA4) expression. Thus, in order to determine the cell-type specific expression of MAP(RA4) and NF-M in the retina, we first carried out a set of experiments to confirm previous observations sustaining that MAP(RA4) expressing cells are postmitotic neuronal precursors (sections *a *and *b *below). Second, in order to determine the cell types in which they are expressed, we performed double-label immunohistochemistry studies for MAP(RA4) or NF-M and markers known to be expressed by the different retinal cell types (Table 1; section *c *below). The ED6 retina was chosen for these studies because all retinal cell types have already begun to differentiate in the central retina [1,25] and MAP(RA4) and NF-M are still broadly expressed in this region of the retina.

### a. MAP(RA4) and NF-M are expressed in postmitotic precursors

**Figure 3:**
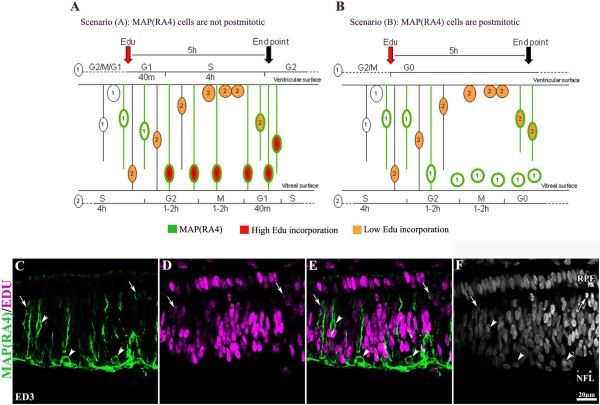
**MAP(RA4) is expressed in postmitotic retinal cells**. (A-B) Schematic representation of two possible scenarios: (A) MAP(RA4) is expressed by non-postmitotic cells; and (B) MAP(RA4) is expressed only by postmitotic cells. Cells numbered with (1) represent the cell population that was at either G2, M or G1, but not S, at the time of EdU delivery (red arrow), while cells numbered with (2) represent cells that were at the last phase of S at the time of EdU delivery. The numbers below S, G2, M and G1 indicate the average length of the corresponding cell cycle steps. The black arrow indicates the time of embryo harvesting and analysis. (C-F) Transverse section of an ED3 retina processed for immunolabeling of MAP(RA4) (green) and EdU incorporation (magenta). MAP(RA4) positive cells can be seen spanning the retina from the ventricular to the vitreal surface (A). Only MAP(RA4) positive cells that are closest to the ventricular surface are EdU positive (C-E; arrows). Arrowheads indicate MAP(RA4) positive/EdU negative cells. NFL: nerve fiber layer; RPE: retina pigmented epithelium.

Previous studies have demonstrated that at developmental stages between ED3 and ED9, the average length for the synthesis phase in retinal progenitor cells is 4 hours, while it takes only 3-5 additional hours to complete the cycle and reach synthesis again, with average times for the remaining cell cycle steps being as follows: G2 = 1-2 h; M = 1-2 h and G1 = 40 min [14,26,27]. Additionally, it has been shown that as short as 20 minutes of exposure to Brdu is enough to clearly label retinal cells at these stages [27], and that MAP(RA4) expression begins within minutes after mitosis has been completed [14]. According to this, in our experimental paradigm consisting of a 5 hour pulse of Edu, two clearly different outcomes should be seen depending on whether MAP(RA4) cells are postmitotic or not. If MAP(RA4) cells are not postmitotic, most MAP(RA4) cells should be colabeled with Edu, particularly those located in the vitreal surface of the retina (Figure 3, A, cell population #1). On the other hand, if MAP(RA4) cells are postmitotic, most if not all MAP(RA4) cells located in the vitreal surface of the retina should be negative for Edu (Figure 3, B, cell population #1). As expected, our analysis demonstrated that most, if not all, MAP(RA4) expressing cells located in the vitreal surface of the retina were negative for Edu, indicating that they had already withdrawn from the cell cycle (Figure 3, C-F; arrowheads). Furthermore, close to 80% of MAP(RA4) expressing cells were not labeled with Edu. The remaining 20% positive for Edu, were located near the ventricular surface of the retina, and most probably represent cells that were at the end of the synthesis phase at the time of Edu delivery and that became postmitotic and expressed MAP(RA4) protein during the 5 hour-pulse window (Figure 3, B; cell population #2; C-F arrows). Similar results were observed for cells expressing NF-M (not shown).

### b. MAP(RA4) and NF-M expressing cells are not Müller cells

**Figure 4:**
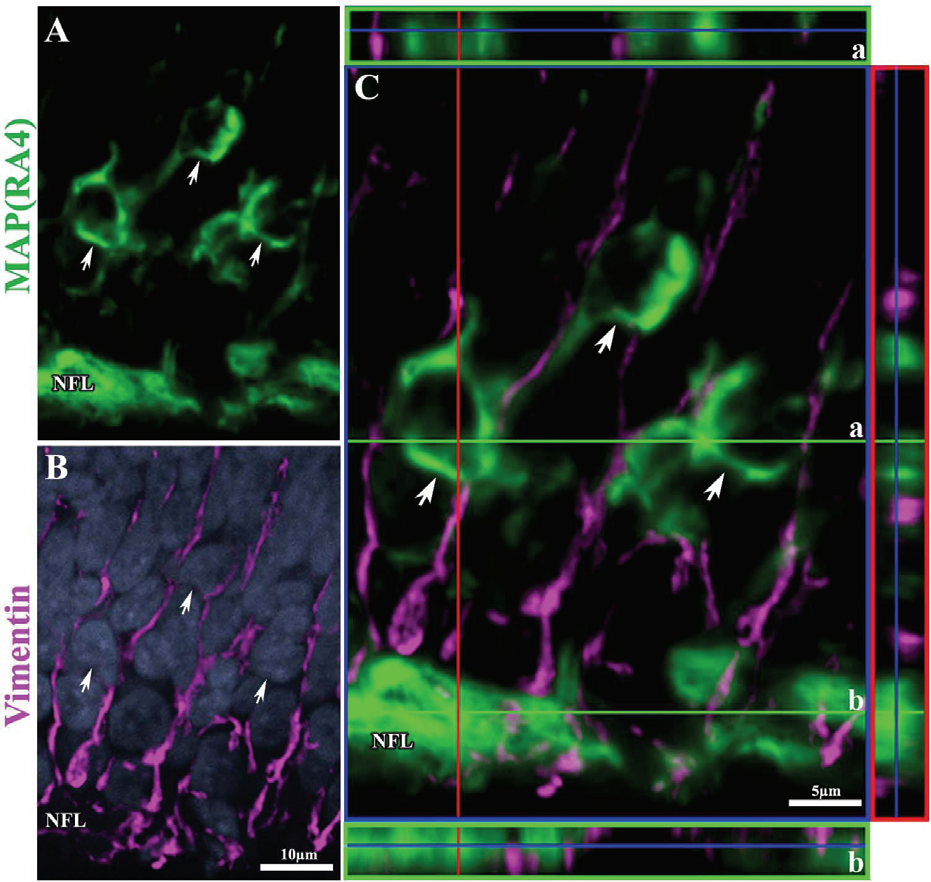
**MAP(RA4) is not expressed in Müller Glial cells**. (A-B) Confocal images of a transverse section of an ED6 retina processed by double-label immunohistochemistry for MAP(RA4) (green; A); and (B) vimentin (magenta) and DAPI nuclear staining (blue). (C) Confocal image with orthogonal views depicting no colocalization of MAP(RA4) and vimentin. Arrows indicate MAP(RA4) positive cells. (a) orthogonal view at the level of MAP(RA4) cell bodies; (b) orthogonal view at the level of the nerve fiber layer (NFL).

Considering that previous studies have suggested that under certain conditions Müller glial cells may be capable of expressing neuronal markers such as the 160 kDa NF-M protein and MAP(RA4) [28], and that the morphology of the cells under study highly resembled that of Müller cells, we decided to test whether MAP(RA4) expressing cells were in fact Müller cells. Colocalization studies for both MAP(RA4) and NF-M (not shown) with vimentin, one of the earliest markers known for Müller cells, showed complete exclusion (Figure 4), demonstrating that MAP(RA4)/NF-M expressing cells during normal development are not Müller cells.

### c. MAP(RA4) and NF-M are expressed in all neuronal retinal precursors

#### Ganglion cells

Several markers known to be expressed by ganglion cell precursors soon after their terminal mitosis were used to correlate ganglion cell fate and MAP(RA4)/NF-M expression. The transcription factor Islet1 has been reported to be specifically expressed in ganglion cells at early stages of development, although as development progresses its expression broadens and Islet1 positive cells can be seen in all the layers of the retina ([29,30]; our own unpublished observations). On the other hand, expression of Hu C/D, an RNA-binding protein expressed in postmitotic neuronal precursors, has been detected in amacrine and ganglion cells in the adult chick and rat retina [31,32]. As shown in Additional File 2, Figure S2, double-label immunohistochemistry with antibodies against Islet1 and Hu C/D and transcription factors specific for retinal cell types other than ganglion cells, demonstrated that at the stages analyzed in this study (ED3-ED6), Islet1 and Hu C/D are expressed almost exclusively in retinal ganglion cells. At these stages, Islet1 positive cells were still mainly localized to the prospective ganglion cell layer (Additonal File 2, Fig S2, A) and were negative for Ap2α, which labels all amacrine cells and a subpopulation of horizontal cells [29,33-35] (Additional File 2, Fig S2, B-D). At ED6, a small fraction of Islet1(+) cells were also positive for Prox1, which at this stage labels migrating horizontal cells [29,35] (Additional File 2, Fig S2, E-H). Hu C/D, on the other hand, labeled a subpopulation of elongated cells scattered throughout the thickness of the retinal neuroepithelium, most probably corresponding to newly postmitotic migrating cells, as well as rounded cells located in the GCL (Additional File 2, Fig S2, I). Hu C/D expression was very similar to that of Islet1, showing no-colocalization with Ap2α while only a few Hu C/D(+) cells were also positive for Prox1 (Additonal File 2, Fig S2, I-P). These cells most likely correspond to the Islet1(+)/Prox1(+) cells described above and thus represent a small proportion of immature differentiating horizontal cells that also express Hu C/D, a phenomenon that has also been observed in the developing rat retina [32]. As expected, at ED6 most if not all Hu C/D(+) cells coexpressed Islet1 (Additional File 2, Fig S2, Q-T).

**Figure 5:**
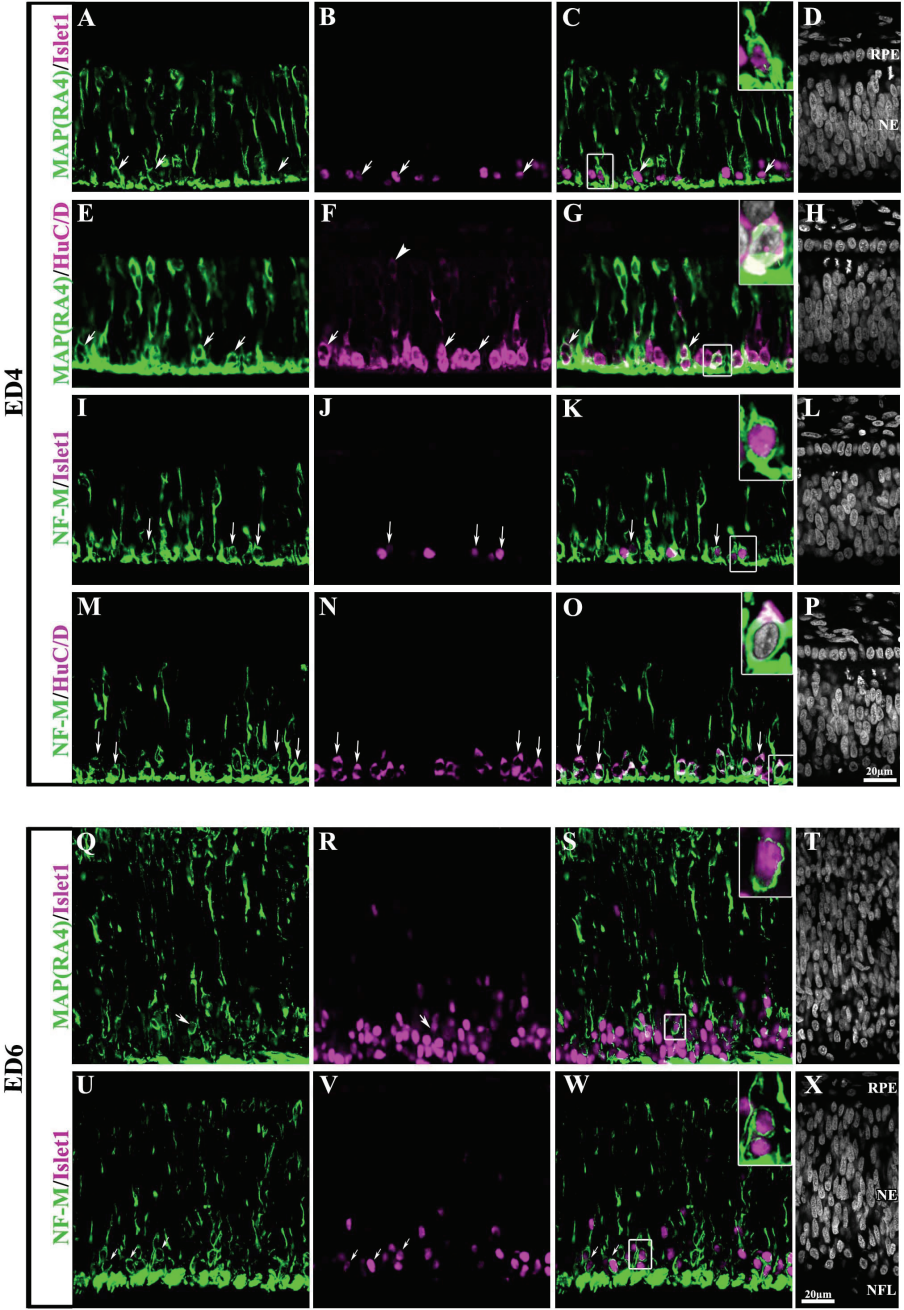
**MAP(RA4) and NF-M are coexpressed in Retinal Ganglion Cells**. Transverse sections of central retina at ED4 (A-P) and ED6 (Q-X) processed for double-label immunohistochemistry for MAP(RA4) (green; A-H and Q-T) or NF-M (green; I-P and U-X) and the ganglion cell markers Islet1 (magenta; A-D, I-L and Q-X) and Hu C/D (magenta; E-H and M-P). At ED4 Islet1 expression is only found in the presumptive GCL (B, J) while Hu C/D expression can be found spanning the neural epithelium with strongest reactivity at the presumptive GCL (F, N). Most Islet1 and Hu C/D positive cells appeared also positive for MAP (RA4) (C, G) and NF-M (K, O). At ED6 MAP(RA4) expression was still observed in a small proportion of Islet1 positive cells (Q-T) while most, if not all, Islet1 positive cells were still positive for NF-M (U-X). Arrows point to colabeled cells. Arrowhead in (F) shows a Hu C/D positive cell on the ventricular side of the neuroepithelium. Insets represent an enlarged image of the boxed-cells in each panel. Scale bar in P applies to A-P and scale bar in × applies to Q-X. NE: neuroepithelium; NFL: nerve fiber layer; RPE: retina pigmented epithelium.

To address whether MAP(RA4) and/or NF-M proteins are expressed by ganglion cell precursors, double-label immunhistochemistry for MAP(RA4) or NF-M with either Islet1 or Hu C/D was carried out at ED4 and ED6. Our results confirmed previous observations showing that newly born migrating ganglion cells, as well as differentiating ganglion cells that have recently reached the vitreal surface of the retina expressed MAP(RA4) (Figure 5, A-H). Similar results were observed for the NF-M protein (Figure 5, I-P). At ED6, MAP(RA4) expression was still observed in a small proportion of Islet1(+) cells (Figure 5, Q-T), while most, if not all, Islet1(+) cells were still positive for NF-M (Figure 5, U-X).

#### Amacrine Cells

**Figure 6:**
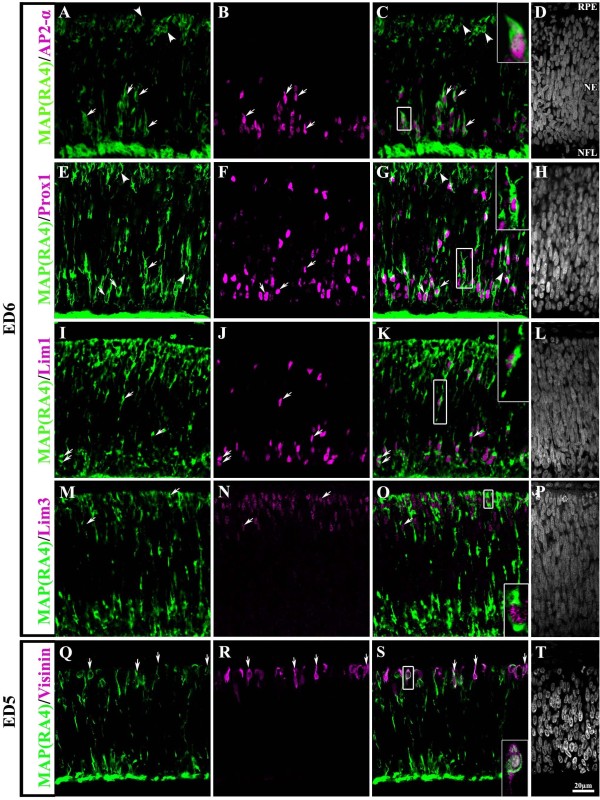
**MAP(RA4) is expressed in all neuronal retinal precursor types**. Transverse sections of central retina at ED6 (A-P) and ED5 (Q-T) processed for double-label immunohistochemistry with MAP(RA4) (green) and markers for different retinal cell types (magenta). MAP(RA4) expression was seen in cells positive for markers specific for amacrine cells (A-D), horizontal cells (E-L), bipolar cells (M-P) and photoreceptors (Q-T). Arrows point to colabeled cells and arrowheads point to MAP(RA4) only cells. Insets are larger images of boxed-cells in their respective panel. NE: neuroepithelium; NFL: nerve fiber layer; RPE: retina pigmented epithelium.

AP2α is a transcription factor expressed in most, if not all, amacrine cells as well as in a subpopulation of horizontal cells early on during their process of differentiation [29,33-35]. At ED6, AP2α(+) cells are mostly located near the vitreal surface of the neuroepithelium, although a considerable number of positive cells can also be seen scattered throughout the neuroepithelium. Double labeling with the MAP(RA4) antibody demonstrated that at this stage, most if not all AP2α(+) cells also expressed MAP(RA4) (Figure 6, A-D; arrows). However, not all MAP(RA4)(+) cells were positive for AP2α (Figure 6, A-D; arrowheads).

#### Horizontal Cells

During retinal development, horizontal cells undergo a process of bi-directional migration. Initially, they migrate from the ventricular side of the neuroepithelium toward the vitreal side, positioning themselves adjacent to the presumptive GCL, where they undergo terminal mitosis at ED6 [35,36]. By st30 (early ED7), they begin to migrate towards the HCL where they arrive by ED8 [35]. To identify horizontal cells in this study we used antibodies against the transcription factors Prox1 and Lim1/2, which label all horizontal cells and a subpopulation of them respectively [29]. Both transcription factors are expressed early in horizontal cell precursors and by ED6, both label cells located on the vitreal side of the retina and some cells that have already started to migrate towards the HCL ([29]; our data). In both cases, MAP(RA4) colabeled cells positive for Prox1 and Lim1 located at various positions throughout the width of the retina (Figure 6, E-L; arrows). Most cells that were either positive for Prox1 or Lim1/2 were also positive for MAP(RA4), but not all MAP(RA4)(+) cells were positive for Prox1 or Lim1 (Figure 6, E-L; arrowheads).

#### Bipolar cells

To visualize bipolar cells, the transcription factor Lim3, which specifically labels this cell population, was used. MAP(RA4) was found to colocalize with most if not all Lim3(+) cells (Figure 6, M-P).

#### Photoreceptor Cells

Photoreceptor cells are born between ED3-7 in the central portion of the retina with roughly 86% of them being cones in the chick [25,37]. Visinin, a calcium binding protein present in photoreceptors and expressed in the chick embryo as early as ED3 [38] was used in this study to analyze whether MAP(RA4) is expressed in photoreceptor precursors. By ED6, visinin(+) cells can be found closely clustered on the ventricular side of the retinal neuroepithelium where the ONL is forming. Therefore, instead of performing the immunohistochemical analysis at ED6, this was done at ED5, when visinin(+) cells are not as tightly clustered, thus making it easier to identify individual cells. Also, similarly to ED6, MAP(RA4)(+) cells at ED5 span the thickness of the retinal neuroepithelium (Figure 6, Q). Double-label immunohistochemistry showed that a significant number of visinin(+) cells were also positive for MAP(RA4) (Figure 6, Q-T).

**Figure 7:**
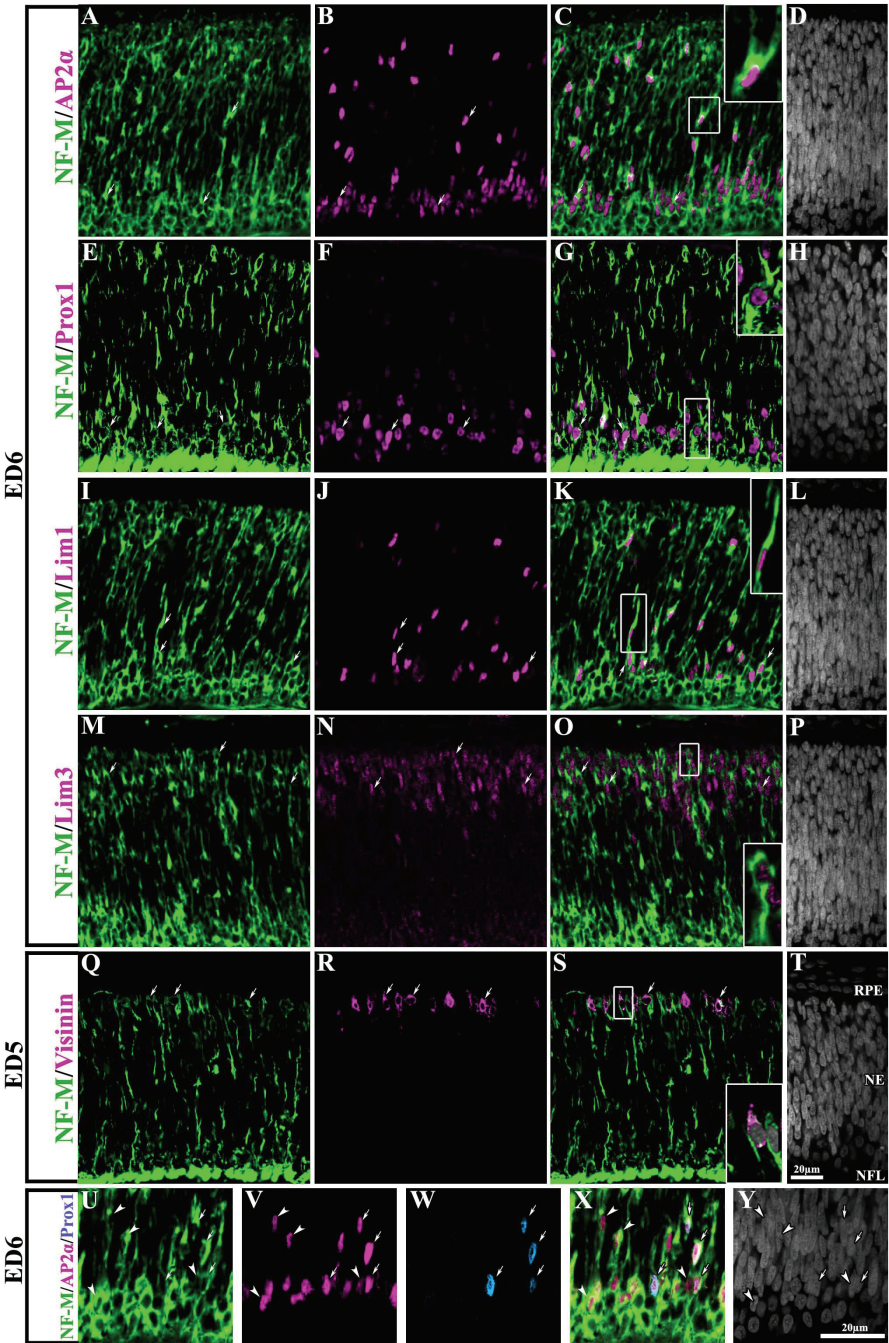
**NF-M is expressed in all neuronal retinal precursor types**. Transverse sections of central retina at ED6 (A-P) and ED5 (Q-T) processed for double-label immunohistochemistry for NF-M (green) and AP2α (A-D), Prox1 (E-H), Lim1/2 (I-L), Lim3 (M-P) and visinin (Q-T) all in magenta. NF-M expression was seen in cells positive for markers specific for amacrine cells (A-D), horizontal cells (E-L), bipolar cells (M-P) and photoreceptors (Q-T). Arrows point to cells that are colabeled. Insets represent magnified images of boxed-cells in their corresponding panel. (U-Y) transverse section of an ED6 retina processed by triple-label immunohistochemistry for NF-M protein (green) in combination with AP2α (magenta) and Prox1 (blue). Arrows point to cells that are triple-labeled while arrowheads indicate cells colabeled only with NF-M and AP2α. Scale bar in T applies to panels A-T and scale bar in Y applies to U-Y. NE: neuroepithelium; NFL: nerve fiber layer; RPE: retina pigmented epithelium.

Similar double labeling studies for NF-M and all cell markers were performed. As in the case of MAP(RA4) expressing cells, cells expressing NF-M were found to coexpress the different retinal cell type markers used in this study (Figure 7, A-T). Since AP2α is known to label not only amacrine cells, but also a subpopulation of horizontal cells, we carried out triple-label immunohistochemistry in order to discriminate between these two populations. Triple labeling for NF-M, AP2α, and Prox1 on ED6 retina showed that the vast majority of NF-M/AP2α(+) cells were amacrine cells (Figure 7, U-Y; arrowheads), while only a small proportion of these cells corresponded to the horizontal cell lineage, as demonstrated by the coexpression of Prox1 (Figure 7, U-Y; arrows). Similar results were obtained with triple labeling for MAP(RA4), AP2α, and Prox1 (not shown).

## Discussion

In this study we have pursued a detailed analysis of the expression of two cytoskeleton components, MAP(RA4) and NF-M, throughout the development of the chick retina. For several years, expression of these two proteins has been broadly used to selectively identify ganglion cells and their committed precursors, in studies using the developing chick retina as a model. Our results, however, demonstrate that contrary to that accepted concept, both proteins are also transiently expressed by precursors of all retinal neuronal cell types. Our main observations can be summarized as follows: (1) Both, MAP(RA4) and NF-M showed a dynamic pattern of expression during retinal development coincident with the progression of retinal cell differentiation; (2) Both proteins were coexpressed spatially and temporally throughout development, although in individual cells NF-M expression appeared before MAP(RA4) and lasted for about 24 hours after MAP(RA4) expression was no longer detected; (3) Both, MAP(RA4) and NF-M were expressed in postmitotic neuronal precursors but not in Müller cell precursors; and (4) Expression of both proteins was seen in ganglion cell precursors and adult differentiated ganglion cells, but they were also transiently expressed by precursors of the photoreceptor, horizontal, bipolar and amacrine cell lineages.

Initial characterization of the RA4 antibody suggested that its corresponding antigen was specifically expressed in ganglion cells and its committed precursors [11]. Although it was stated that the possibility of RA4 antibody labeling other cell types besides ganglion cells could not be completely ruled out, it was concluded that the labeled cells could be considered as belonging exclusively to the ganglion cell lineage. This conclusion was based principally in its temporal and spatial expression at the stages of development analyzed, the morphology of the labeled cells, and the fact that in the mature retina RA4 signal is detected only in the ganglion cell axons [11,14]. Our observations on the spatial and temporal expression of the MAP(RA4) antigen agreed for the most part with this initial characterization, but some important differences were seen. First, our studies showed significant MAP(RA4) labeling of radially oriented cells in the central region of the retina still present at ED6, while the studies from McLoon and Barnes [11] described lack of staining in this region at this time of development. These differences could be easily explained by variations in the stages of the embryos utilized. Embryos at ED6 could range anywhere from stage 27 to 31, highly overlapping with developmental stages on ED7 (30 to 33; [16]). As shown in results, retinas from embryos at stage 31 of development, which can be found either at ED6 or ED7, did indeed show lack of MAP(RA4) labeling in radially oriented cells in the central region of the retina. In contrast, retinas from embryos at stage 29 (the average stage at ED6) consistently showed persistence of radially-oriented MAP(RA4) positive cells in the central retina. Furthermore, our observations are also supported by those from Moreira and Adler [39], reporting MAP(RA4) expression in radially oriented cells in the central region of the retina at ED6. Therefore, in this study we have systematically used embryos at stage 29, which is found only at ED6 [16] and which is one of the stages of development most commonly used in studies addressing differentiation of retinal progenitor cells in the chick. Second, we also consistently observed MAP(RA4) labeling in cells located in the horizontal cell layer, which were not reported by McLoon et al [11]. In agreement with our observations, other authors have also seen MAP(RA4) signal in the horizontal cell layer, although it was not necessarily reported as labeling horizontal cells [12,15,28,39].

As in the case of the RA4 antibody, initial characterization of the pattern of expression of the chick NF-M in the retina, was also interpreted as being exclusively expressed by ganglion cells and its precursors, based on its temporal and spatial distribution and the similarity of its expression pattern to that of RA4 [12,13,15,40]. In both cases, one of the main elements considered to conclude that the labeled cells were exclusively GC precursors was the time of development analyzed, since according to the information available at the time, it was assumed that the majority, if not the only, cells being generated at those developmental times were GCs and therefore GC precursors were the only ones migrating from the ventricular to the vitreal surface [11-14,40]. However, birthdating studies and immunohistochemical analysis of transcription factors specific for different retinal cell types have afterward consistently demonstrated that as early as ED3 other cell types besides ganglion cells are already beginning to differentiate [25,29,35,36,41]. Furthermore, amacrine and horizontal cells are also known to migrate towards the vitreal surface, and their timing of migration highly overlaps with that of the GC precursors [29,33,35,36,42]. Further supporting our observations, studies in the developing mouse retina have shown that the NF-M protein is also expressed in cells other than GCs, including amacrine and horizontal cells [43-45].

The current availability of well characterized antibodies that recognize transcription factors and other proteins expressed in specific retinal cell types, either exclusively or limited to just a few cell lineages, allowed us to re-address the expression pattern of MAP(RA4) and NF-M during normal retinogenesis. To the best of our knowledge, our study is the first to analyze the expression of these two molecules in combination with an array of proteins that allows unambiguous identification of all retinal cell type precursors. Thus, our results clearly show that expression of both, MAP(RA4) and NF-M, is not restricted to the ganglion cell lineage, but is also transiently expressed by all neuronal retinal precursor types. An increasing body of information suggests that phosphorylation of the NF subunits and its interactions with MAPs are important for cellular processes involved in cell differentiation such as migration, establishment of cell morphology and axonal growth (reviewed by [4,8,46]). Furthermore, these mechanisms seem to be of particular importance for the stabilization of the stationary, slow turn-over cytoskeletal network present in the GC axons and other long axon-bearing neurons [3,4,8,46]. We therefore propose that MAP(RA4) and phosphorylated NF-M proteins may be present in all postmitotic neuronal retinal precursors as part of the mechanisms regulating their migration and early morphological differentiation. Once these cellular processes have been accomplished, expression of MAP(RA4) and phosphorylated NF-M stops, or decreases to undetectable levels, in most neuronal cell types except for long-axon projecting neurons such as bullwhip cells (which retain NF-M expression) and ganglion cells (which retain expression of both MAP(RA4) and NF-M). In these cells, high levels of expression of these proteins may become permanent to ensure the development and maintenance of the stationary axonal cytoskeleton network.

The implications of our findings are of particular relevance for the interpretation of previous studies of retinal neurogenesis in which expression of both, MAP(RA4) and NF-M have been used as markers for ganglion cells exclusively. Some examples of such cases are discussed here. Several studies directed to investigate the role of the Notch/Delta signaling pathway in regulating ganglion cell fate determination were carried out by using gain- and loss-of function approaches in the developing chick retina, and the use of MAP(RA4) and NF-M (and in some cases also Islet1) to identify differentiating GCs [47-51]. These studies were mostly done in retinas from ED3-ED6, when GCs are actively differentiating [47-49,51] but also in some cases at later stages (ED7-11) when GC genesis has ceased to occur [50]. The main conclusions from these studies were that a decrease in Notch/Delta pathway leads to an increase in number and premature differentiation of ganglion cells, as well as novel production of GCs later than the normal window of GC genesis, while an over-activation of the pathway conversely leads to a decrease in the number of GCs. Additionally, based on the expression of MAP(RA4) and NF-M, Austin and collaborators concluded that all retinal progenitor cells are competent to become GCs and that GC precursors selectively arise from the pool of competent cells by the action of Notch [47]. In contrast, our findings demonstrating that during neurogenesis in the chick retina MAP(RA4) and NF-M expression is not confined to the GC lineage, rather suggest that the observations discussed above most probably represent a more general role for the Notch/Delta pathway in retinal neurogenesis. In light of our results, the MAP(RA4) and/or NF-M positive populations analyzed in these studies may actually represent a postmitotic cell population containing different cell-type precursors. Similar considerations apply to the use of Islet1, which is known to be expressed in other cell types besides GCs, even at the stages of development used in these studies ([29,36] our own study). Thus, the changes observed in the number of cells expressing these markers, which were interpreted as selective effects of Notch/Delta signaling in GC fate determination, may in fact represent a combined increase or decrease of differentiating cells of several lineages, or even a delayed or premature differentiation of some cell types other than GCs. This scenario is indeed supported by studies from Henrique et al [30] and Kubo et al [52] showing that in the chick retina manipulation of the Notch/Delta pathway influences the differentiation of most if not all neuronal retinal cell lineages. Further support comes from experiments done in Xenopus, where over-activation of the Notch/Delta system induces an increase in retinal progenitor cells with a concomitant decrease in the number of all differentiated neuronal cell types [53,54] and those in mice in which inhibition of this pathway induced an increase in the number of photoreceptors at the expense of all other cell types [55,56].

MAP(RA4) and NF-M have also been commonly used as markers of GCs in investigating the potential of RPE to transdifferentiate into neuronal cells [57-64]. Several of these studies were aimed at testing the ability of different molecules to specifically induce transdifferentiation of RPE towards a GC phenotype [59-61,63]. For example, in experiments directed to test the ability of the transcription factors Cath5 and NSCL1 to induce this phenomenon, Ma et al [60] and Xie et al [61], concluded that these transcription factors might be necessary for the commitment and further differentiation of GC precursors initially induced in the presence of FGF. This conclusion was based on the expression of MAP(RA4), NF-M and a few other markers expressed by GCs -although not exclusively by them- such as calretinin, MAP2 and Islet1. However, in these experiments MAP(RA4) positive cells consistently failed to express other transcription factors specific for GCs such as Brn3a or NeuN, and in some cases even Islet1. A possible interpretation of these results is that even though Cath5 and NCSL1 may be capable of inducing RPE cells to adopt a GC fate they may not be sufficient to support their further differentiation. However, when considered on the context of the results presented in this study, an alternative scenario arises, where the increase in MAP(RA4) and/or NF-M positive cells may reflect a more general effect of transdifferentiation into a neuronal-like phenotype, rather than specific transdifferentiation into GCs.

Our findings also demonstrate that MAP(RA4) and NF-M may be useful for the specific identification of all post-mitotic neuronal precursors during retinal differentiation. This is of particular importance, since to the best of our knowledge, there are no other molecules characterized so far that could be used to generally identify postmitotic neuronal retinal precursors from the pool of mitotically active progenitors and/or the differentiated cell population during retinogenesis. It is important to notice, however, that this may be applicable only to developmental stages after the establishment of the optic cup, since NF-M and MAP(RA4) are also transiently expressed in proliferating neuroepithelial cells in several regions of the brain, including the optic vesicle neuroepithelium, during early embryonic development (ED1-2.5) ([40]; our own unpublished observations). Although it would be of interest to investigate with more detail the expression pattern of these two molecules during the transition from the optic vesicle to the optic cup, such analysis is beyond the scope of this study.

## Conclusions

Our results demonstrate that MAP(RA4) and NF-M are not restricted to GCs, but rather transiently expressed by all retinal neuronal cell types in a developmentally-regulated manner. We propose that initial expression of these molecules may be important in all retinal neuronal cell types for cellular processes involved in cell differentiation such as migration, cell morphology and axonal growth, while high sustained levels of expression may be necessary for overt differentiation of long-projecting axons cell types including GCs and bullwhip cells, and for maintenance of the axonal cytoskeleton network in their adult state. Our findings also demonstrate that MAP(RA4) and NF-M may be useful for the identification of post-mitotic precursors from the pool of mitotically active progenitors and/or the differentiated cell population during retinogenesis. Retrospectively, although the conclusions from previously published studies using these proteins as specific markers for GC precursors may still be valid, our results suggest that they may need to be re-evaluated.

## Competing interests

The authors declare that they have no competing interests.

## Authors' contributions

CG, MM and MVC-S designed and performed experiments and analyzed the data. CG and MVC-S wrote the paper and MM contributed with critical reading and editing of the text. All authors read and approved the final manuscript.

## Supplementary Material

Additional File 1 **Figure S1. Spatial and temporal expression pattern of MAP(RA4) and NF-M**. (A) At ED3 MAP(RA4) and NF-M expression is restricted to radially-oriented cells in the central portion of the retina neuroepithelium. At this stage, transient expression of both markers can also be seen in the lens (L). (B) At ED6, MAP(RA4) and NF-M expression in radially-oriented cells has progressed toward the periphery, spanning most of the retina. (C) As the retina matures, both markers show a central-low/periphery-high gradient of expression. At ED8, radially-oriented cells are seen only in peripheral retina (c1 and c3). On the other hand, the central portion of the retina shows MAP(RA4) and NF-M expression restricted to the HCL, IPL GCL and NFL (c2 and c4). Asterisks indicate nerve fibers outside of the eye that are also positive for MAP(RA4) and NF-M. GCL: ganglion cell layer; HCL: horizontal cell layer; IPL: inner plexiform layer; L: Lens; NFL: nerve fiber layer. Click here for file

Additional File 2 **Figure S2. Islet1 and Hu C/D are almost exclusively expressed in ganglion cells at developmental stages corresponding to ED3-ED6**. Transverse sections of central retina at ED6 processed by double-label immunohistochemistry for Islet1 (A-H) or Hu C/D (I-P) and AP2α (A-D; I-L) or Prox1 (E-H; M-P). Neither Islet1 nor Hu C/D positive cells colabeled with Ap2α (A-D; I-L), and in both cases only a small proportion of them appeared colabeled with Prox1 (E-H; M-P). (Q-T) Double-label immunohistochemestry for Hu C/D (green) and Islet1 (magenta). Most if not all Islet1(+) cells were also positive for Hu C/D (arrows). Scale bar in H applies to A-H and scale bar in T applies to I-T. NE: neuroepithelium; GCL: ganglion cell layer. Click here for file
