# Supplementary figures and images for: Cytoskeleton proteins previously considered exclusive to Ganglion Cells are transiently expressed by all retinal neuronal precursors
Source: BMC Dev Biol. 2011 Jul 22;11:46. doi: 10.1186/1471-213X-11-46 (PMC3161946; doi:10.1186/1471-213X-11-46)

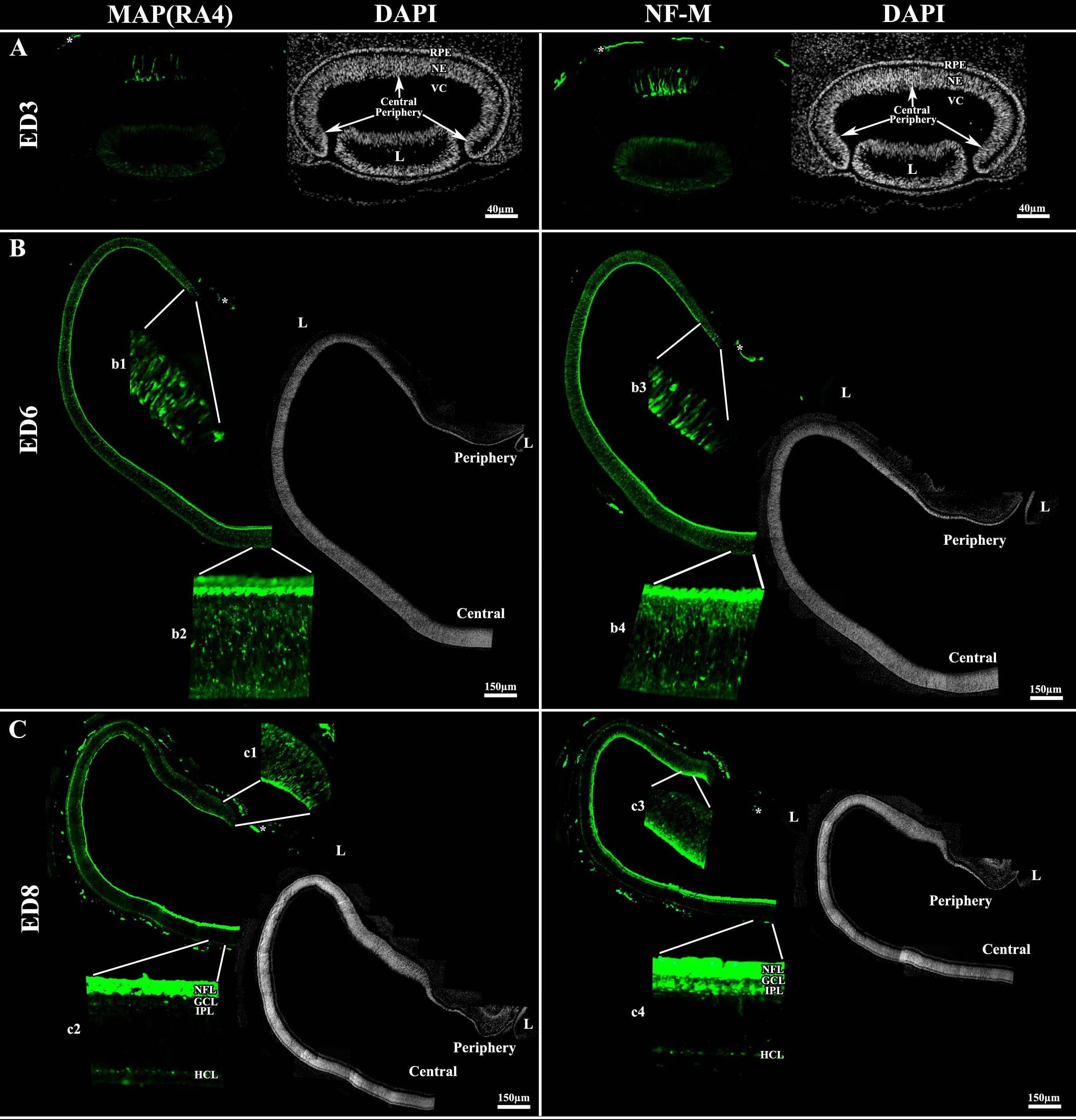

Supplement: Additional File 1 — Figure S1. Spatial and temporal expression pattern of MAP(RA4) and NF-M. (A) At ED3 MAP(RA4) and NF-M expression is restricted to radially-oriented cells in the central portion of the retina neuroepithelium. At this stage, transient expression of both markers can also be seen in the lens (L). (B) At ED6, MAP(RA4) and NF-M expression in radially-oriented cells has progressed toward the periphery, spanning most of the retina. (C) As the retina matures, both markers show a central-low/periphery-high gradient of expression. At ED8, radially-oriented cells are seen only in peripheral retina (c1 and c3). On the other hand, the central portion of the retina shows MAP(RA4) and NF-M expression restricted to the HCL, IPL GCL and NFL (c2 and c4). Asterisks indicate nerve fibers outside of the eye that are also positive for MAP(RA4) and NF-M. GCL: ganglion cell layer; HCL: horizontal cell layer; IPL: inner plexiform layer; L: Lens; NFL: nerve fiber layer. [file 1471-213X-11-46-S1.JPEG]

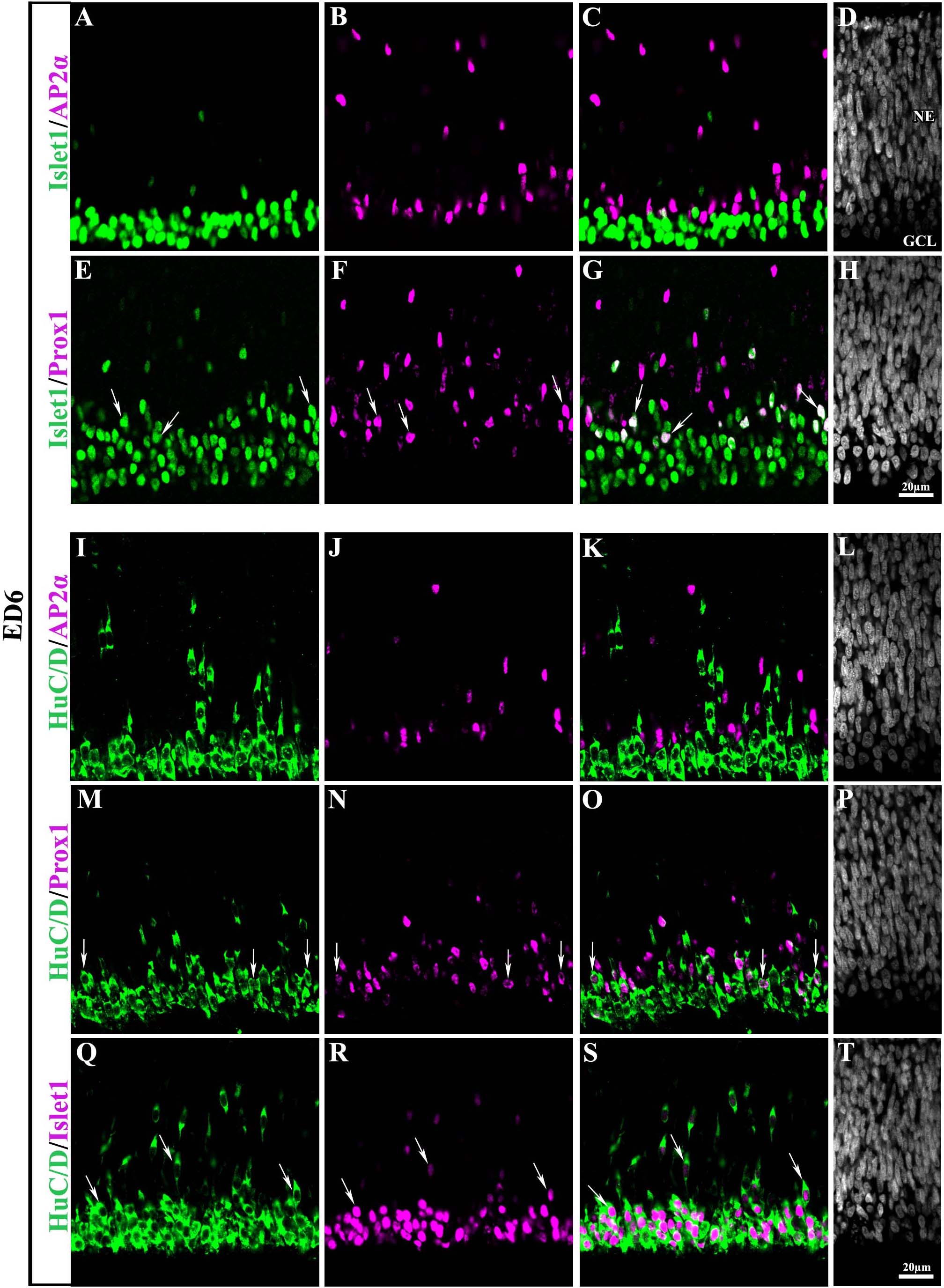

Supplement: Additional File 2 — Figure S2. Islet1 and Hu C/D are almost exclusively expressed in ganglion cells at developmental stages corresponding to ED3-ED6. Transverse sections of central retina at ED6 processed by double-label immunohistochemistry for Islet1 (A-H) or Hu C/D (I-P) and AP2α (A-D; I-L) or Prox1 (E-H; M-P). Neither Islet1 nor Hu C/D positive cells colabeled with Ap2α (A-D; I-L), and in both cases only a small proportion of them appeared colabeled with Prox1 (E-H; M-P). (Q-T) Double-label immunohistochemestry for Hu C/D (green) and Islet1 (magenta). Most if not all Islet1(+) cells were also positive for Hu C/D (arrows). Scale bar in H applies to A-H and scale bar in T applies to I-T. NE: neuroepithelium; GCL: ganglion cell layer. [file 1471-213X-11-46-S2.JPEG]
